# Hoof, Vaginal and Milk Microbiota Alterations in Dairy Cows with Foot Rot

**DOI:** 10.3390/ani16060920

**Published:** 2026-03-15

**Authors:** Pengyu Zhao, Kai Jiang, Haitao Sun, Xianjing He, Donghua Guo

**Affiliations:** 1 College of Animal Science and Veterinary Medicine, Heilongjiang Bayi Agricultural University, Daqing 163319, China; zhaopengyu_zoey@163.com (P.Z.); jk865789845@163.com (K.J.); htsunoo@163.com (H.S.); xianjinghe@126.com (X.H.); 2 China Key Laboratory of Bovine Disease Control in Northeast China, Ministry of Agriculture and Rural Affairs, Daqing 163319, China; 3 Heilongjiang Provincial Key Laboratory of Prevention and Control of Bovine Diseases, Daqing 163319, China

**Keywords:** foot rot, milk microbiota, vaginal microbiota, *Fusobacterium necrophorum*

## Abstract

Foot rot is a major infectious cause of lameness in dairy cows and can severely compromise production performance and animal welfare. However, it remains unclear whether foot rot is accompanied by microbiota alterations beyond the hoof, particularly in the vagina and milk. We conducted a case–control study of 24 Holstein cows (12 with clinical foot rot and 12 healthy controls). Hoof swabs, milk, and vaginal swabs were collected and analyzed using 16S rRNA gene amplicon sequencing, alongside quantitative PCR. Microbial community composition differed between cows with foot rot and healthy controls across hoof (R^2^ = 0.26), vaginal (R^2^ = 0.072), and milk (R^2^ = 0.16) samples (Bray–Curtis, PERMANOVA, *p* < 0.001). Simpson diversity was lower in the hoof and vaginal microbiota (*p* < 0.05) but not in milk (*p* = 0.13). Differential abundance analysis identified enrichment of *Fusobacterium* in hoof and vaginal samples (*p* < 0.001), and absolute qPCR confirmed a higher vaginal load of *Fusobacterium necrophorum* in cows with foot rot (*p* < 0.05). Milk samples from foot rot cows showed enrichment of *Clostridium* and *Porphyromonas*. Co-occurrence network analysis revealed reduced microbial connectivity in foot rot-affected cows (average degree: 4.98 vs. 6.70) and increased network fragmentation (14 vs. 9 modules), suggesting lower ecosystem stability. Overall, foot rot was associated with microbiota shifts across the hoof, vagina, and milk, highlighting the potential value of integrated management strategies that consider extra-hoof microbial alterations.

## Simple Summary

Foot rot is a common infectious hoof disease that causes pain and lameness in dairy cows and can reduce milk production and overall performance. This study aimed to determine whether cows with foot rot show changes in the bacterial communities not only on the hoof but also in the udder and the vagina. Our results showed that foot rot was associated with clear shifts in the bacteria found in hoof swabs, vaginal swabs, and milk. In affected cows, the abundance of anaerobic bacteria and other potentially harmful bacteria increased, and the stability of the bacterial community decreased. These findings suggest that foot rot may not be only a local hoof problem and that herd management should consider its broader effects on cow health. Strengthening prevention and early control of hoof disease, while paying attention to udder health and reproductive health, may help improve animal welfare and farm productivity.

## 1. Introduction

Lameness is a prevalent and detrimental health issue in intensive dairy farming that reduces milk production and impairs reproductive performance, resulting in substantial economic losses for the dairy industry [1,2]. Persistent pain and stress associated with lameness activate the hypothalamic–pituitary–adrenal axis and suppress reproductive function [3,4]. Additionally, lame cows often reduce feed intake, contributing to negative energy balance and, consequently, decreased milk yield and reproductive efficiency [5]. However, these explanations primarily emphasize neuroendocrine regulation and energy metabolism and do not fully clarify the molecular mechanisms or specific pathways linking lameness to production losses.

Recent microbiota research offers a new perspective on this issue. Growing evidence suggests that microbial communities across host anatomical sites may be interconnected via immune modulation and metabolite exchange, forming an integrated network [6,7]. Dysbiosis at one site may propagate and influence microbial balance at other sites, potentially contributing to complex disease phenotypes [8]. Within this framework, vaginal and mammary microbiota are crucial factors influencing reproductive and lactation performance. Studies based on 16S rRNA sequencing show that the vaginal and milk microbiota of healthy cows have stable diversity and characteristic bacterial profiles [9,10]. Disruption of this balance and the enrichment of opportunistic pathogens have been linked to decreased conception rates and increased mastitis risk [11,12]. Therefore, the composition of the vaginal and mammary microbiota may reflect the local health status and may also serve as a potential biomarker for assessing reproductive and lactation performance, offering an important avenue for exploring microbial mechanisms underlying production losses associated with lameness.

Foot rot, a major cause of infectious lameness, is highly contagious in wet, unsanitary environments, with prevalence often reported to reach 20–30% in high-yielding intensive dairy herds [13]. This condition is usually caused by polymicrobial infections, primarily involving *Fusobacterium necrophorum* and *Dichelobacter nodosus*, often accompanied by *Treponema* species [14,15]. These infections result in severe interdigital tissue damage, significantly disrupting local microbial ecology. Notably, *F. necrophorum* is not restricted to the hoof and has been detected in other ecological niches, including the rumen, reproductive tract, and feces [16]. More importantly, during specific physiological periods such as the peripartum and postpartum stages, reproductive tract pathogens including *F. necrophorum* have been detected in peripheral blood [17], and a potential for hematogenous dissemination has been proposed in previous studies.

Although the negative impacts of foot rot on production performance have been widely reported, it remains unclear whether these effects are mediated by alterations in vaginal and mammary microbiota. To address this gap, we used 16S rRNA gene amplicon sequencing to compare the composition and diversity of hoof, vaginal, and milk microbiota between healthy cows and cows with foot rot. Additionally, absolute quantitative PCR was used to detect the distribution of *F. necrophorum* across different anatomical sites. We hypothesized that foot rot is accompanied by local hoof microbial dysbiosis and may also coincide with compositional shifts in the vaginal and milk microbiota. We further postulated that these multi-site microbiota alterations may be associated with differences in reproductive and lactation performance observed in lame cows. This study aimed to elucidate the association between foot rot and microbial imbalance at distant sites from a microbial ecology perspective, providing evidence to inform comprehensive foot rot control and future microbiota-targeted interventions.

## 2. Materials and Methods

### 2.1. Study Design and Animals

This matched case–control study was conducted at a large commercial Holstein dairy farm in Jixi City, Heilongjiang Province, China. All cows enrolled in this study were privately owned by the participating farm and were selected from the milking herd according to the inclusion and exclusion criteria described below. The farm employed a free-stall housing system with more than 2000 lactating cows managed under standardized management, including total mixed ration (TMR) feeding, ad libitum access to water, routine vaccination, and regular hoof health monitoring. All experimental protocols were reviewed and approved by the Ethics Committee of Heilongjiang Bayi Agricultural University (Approval No. DWKJXY2022071).

### 2.2. Enrollment

Cows were first screened for lameness during routine herd health monitoring using the 5-point locomotion scoring system described by Sprecher et al. [18]. (1 = normal gait, 2 = mildly abnormal gait, 3 = moderately lame with an arched back, 4 = lame with one or more limbs favored, and 5 = severely lame or non-weight bearing). Cows with locomotion scores ≥ 3 were then examined by veterinarians. Foot rot was clinically suspected in cows showing interdigital skin lesions (swelling, necrosis, or ulceration) accompanied by foul-smelling purulent exudate, and cows with concurrent hoof disorders (digital dermatitis, laminitis, or white line disease) were excluded.

For laboratory confirmation, exudate from deep interdigital lesions was collected using sterile swabs, and the presence of *F. necrophorum* was confirmed by PCR targeting the leukotoxin A (*LktA*) gene using primers *F. necrophorum LktA*-F (5′-ACAATCGGAGTAGTAGGTTC-3′) and *F. necrophorum LktA*-R (5′-ATTGGTAACTGCCACTGC-3′). Reactions were performed in 12.5 μL using a 2× Taq Master Mix (Novoprotein, Shanghai, China), with 0.2 μL (10 μM) of each primer and 1 μL of template DNA; nuclease-free water was added to volume. Amplification was carried out on a C1000 Touch Thermal Cycler (Bio-Rad, Hercules, CA, USA) under the following conditions: 94 °C for 5 min; 35 cycles of 94 °C for 30 s, 59 °C for 30 s, and 72 °C for 30 s; and 72 °C for 5 min. Only *LktA*-positive cows were enrolled as cases.

For each confirmed foot rot case, parity, days in milk (DIM), daily milk yield, locomotion score, somatic cell count (SCC), body condition score (BCS), and vaginal discharge characteristics were recorded. Healthy control cows were selected from the same farm. One healthy control cow was matched to each case based on parity (±1 lactation) and DIM (±7 days). Controls were required to have a normal gait (locomotion score = 1), no current or historical hoof disorders, no clinical mastitis, SCC < 200,000 cells/mL, and normal vaginal discharge. Control cows underwent the same clinical assessments and metadata collection as cases. A total of 12 foot rot cases and 12 matched controls were enrolled. For both healthy controls and foot rot cows, farm medication records confirmed no antibiotic use within 14 days prior to sampling, and all foot rot cows were sampled before receiving any treatment.

### 2.3. Sample Collection

On the day of clinical diagnosis, hoof, vaginal, and milk samples were collected from each enrolled cow using aseptic techniques. After proper restraint, the affected hooves were rinsed with clean water, and sterile swabs were collected from the interdigital lesion exudate. For vaginal sampling, the vulva was first cleaned with water to remove feces and debris and then disinfected with 75% ethanol. A sterile disposable swab was gently inserted approximately 15 cm into the vagina, held in place for 5 s, and withdrawn. For milk sampling, teats were disinfected with an iodophor-based solution, and disposable gloves were worn. The first three streams were discarded, and milk samples were collected into sterile 15 mL centrifuge tubes. Field blanks were included during sampling to monitor environmental contamination. An empty sterile tube for milk processing and air-exposed sterile swabs for vaginal and hoof sampling were processed in parallel with the corresponding samples. Samples from foot rot cows were labeled F (hoof swab), FV (vaginal swab), and FM (milk), and those from healthy cows were labeled HF, HV, and HM, respectively. Multiple samples were collected and immediately placed in a portable cryogenic container for transport and maintained at −80 °C until analysis.

### 2.4. DNA Extraction and 16S rRNA Gene Amplicon Sequencing

Genomic DNA was extracted from hoof swabs, vaginal swabs, and milk samples using a commercial kit (Omega Bio-Tek, Norcross, GA, USA) according to the manufacturer’s instructions. DNA concentration and purity were assessed using a NanoDrop NC2000 spectrophotometer (Thermo Fisher Scientific, Waltham, MA, USA). DNA integrity was evaluated by electrophoresis on 1% agarose gels, and gels were visualized using an Amersham Imager 600 system (GE Healthcare, Chicago, IL, USA).

The V3–V4 region of the bacterial 16S rRNA gene was amplified using primers 338F (5′-ACTCCTACGGGAGGCAGCA-3′) and 806R (5′-GGACTACHVGGGTWTCTAAT-3′) with unique 7 bp barcodes for multiplexing. PCR amplification was performed in 25 μL reactions containing 1 μL template DNA, 1 μL (10 μM) of each primer, 5 μL 5× buffer, 2 μL dNTPs (2.5 mM), 0.25 μL FastPfu DNA polymerase (5 U/μL), and nuclease-free water to volume. Thermal cycling was: 98 °C for 3 min; 27 cycles of 98 °C for 30 s, 52 °C for 30 s, and 72 °C for 45 s; and a final extension at 72 °C for 5 min. Amplicons were purified using VAHTS™ DNA Clean Beads (Vazyme, Nanjing, China) and quantified with the Quant-iT PicoGreen dsDNA Assay Kit (Invitrogen, Carlsbad, CA, USA). Equimolar amplicons were pooled for library construction, and paired-end sequencing (2 × 250 bp) was performed on an Illumina NovaSeq 6000 platform (Shanghai Personal Biotechnology Co., Ltd., Shanghai, China) using the NovaSeq 6000 SP Reagent Kit. Negative controls were included throughout DNA extraction and PCR amplification. Extraction blanks and no-template controls were processed alongside samples. Raw reads are available in GSA under accession CRA063243 (BioProject: PRJCA057974).

### 2.5. Absolute Quantitative PCR for Fusobacterium necrophorum

The abundance of *F. necrophorum* in vaginal samples (FV and HV) was quantified by absolute quantitative PCR (qPCR). To construct a plasmid standard, the *LktA* gene fragment was amplified using primers 5′-ATGAGCGGCATCAAAAATAACGTTCAG-3′ (forward) and 5′-ACCGTAATGCTTCCATTCGGATTCAAT-3′ (reverse), and the amplicon was cloned into the pMD18-T vector. Absolute qPCR was performed using *LktA*-specific primers 5′-AACGTTCAGAGGACAAGGAAGAG-3′ (forward) and 5′-CCTCACCGTCATAGTGTTAATCA-3′ (reverse). Ten-fold serial dilutions of the recombinant plasmid (10^2^–10^7^ copies/μL) were prepared to generate the standard curve.

Real-time PCR was conducted on a QuantStudio™ 3 Real-Time PCR System (Thermo Fisher Scientific, Waltham, MA, USA) using SYBR Green-based detection (2× SYBR^®^ Premix Ex Taq; Takara Bio, Dalian, China). Reactions were run in a final volume of 12.5 μL containing the SYBR master mix, primers, and 1 μL of template DNA, with nuclease-free water added to volume. The cycling program consisted of 95 °C for 30 s, followed by 40 cycles of 95 °C for 5 s and 60 °C for 30 s, with subsequent melt-curve analysis to verify specificity. *F. necrophorum* copy numbers were calculated from the plasmid standard curve (y = −2.9436x + 27.834, R^2^ = 0.9976; *p* = 0.0024).

### 2.6. Bioinformatics and Statistical Analysis

Microbiome sequencing data were analyzed primarily using QIIME 2 and R (version 4.3.3). Alpha diversity was assessed using the Chao1 richness index and the Simpson diversity index, and differences between groups were evaluated with the Wilcoxon rank-sum test. Beta diversity was calculated based on Bray–Curtis dissimilarities, and differences in community structure were tested by permutational multivariate analysis of variance (PERMANOVA). Differences in taxon abundance between groups were assessed using linear discriminant analysis effect size (LEfSe), with an LDA score > 3 and a Wilcoxon test *p* value < 0.05 as the significance thresholds. *p*-values from taxon-level differential abundance analyses (phylum and genus) were adjusted for multiple comparisons using the Benjamini–Hochberg false discovery rate (BH-FDR) procedure, and q < 0.05 was considered statistically significant. The co-occurrence network analysis was performed using SparCC (Friedman and Alm, 2012) analysis (|r| > 0.6 and FDR < 0.05) and visualized by Gephi (v 0.10.1). The roles of the network nodes were defined according to within-module connectivity (Zi) and among-module connectivity (Pi) [19]. Based on Zi and Pi values, nodes were categorized into network hubs, module hubs, connectors, and peripherals (Zi > 2.5 and Pi > 0.62; Zi > 2.5 and Pi < 0.62; Zi < 2.5 and Pi > 0.62; Zi < 2.5 and Pi < 0.62) [20]. Absolute qPCR for *F. necrophorum* analysis between the two groups was performed using a paired Student’s *t*-test (GraphPad Prism 8.0). For single comparisons, *p* < 0.05 was considered statistically significant; for multiple taxon-level comparisons, statistical significance was assessed using BH-FDR-adjusted q-values (q < 0.05).

## 3. Results

### 3.1. Baseline Characteristics of Study Animals

**Figure 1:**
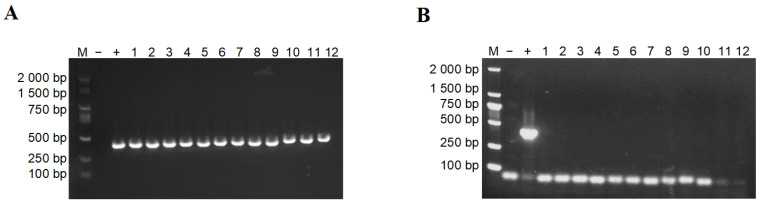
PCR detection of *F. necrophorum* in hoof samples. (**A**) Cows with foot rot. (**B**) Healthy cows. Lane M, DNA size marker; lane −, negative control; lane +, positive control; lanes 1–12, individual hoof samples.

Groups were well-matched for parity and days in milk. Foot rot cows had significantly lower milk yield compared to controls; somatic cell count and body condition score did not differ significantly between groups (Table 1). All hoof samples from foot rot cows tested positive for *F. necrophorum*, as confirmed by PCR targeting the *LktA* gene. Gel electrophoresis revealed specific bands at approximately 402 bp (Figure 1A), confirming the presence of the pathogen. In contrast, no *F. necrophorum* was detected in hoof samples from healthy cows (Figure 1B).

### 3.2. Alterations in Hoof Microbiota of Dairy Cows with Foot Rot

**Figure 2:**
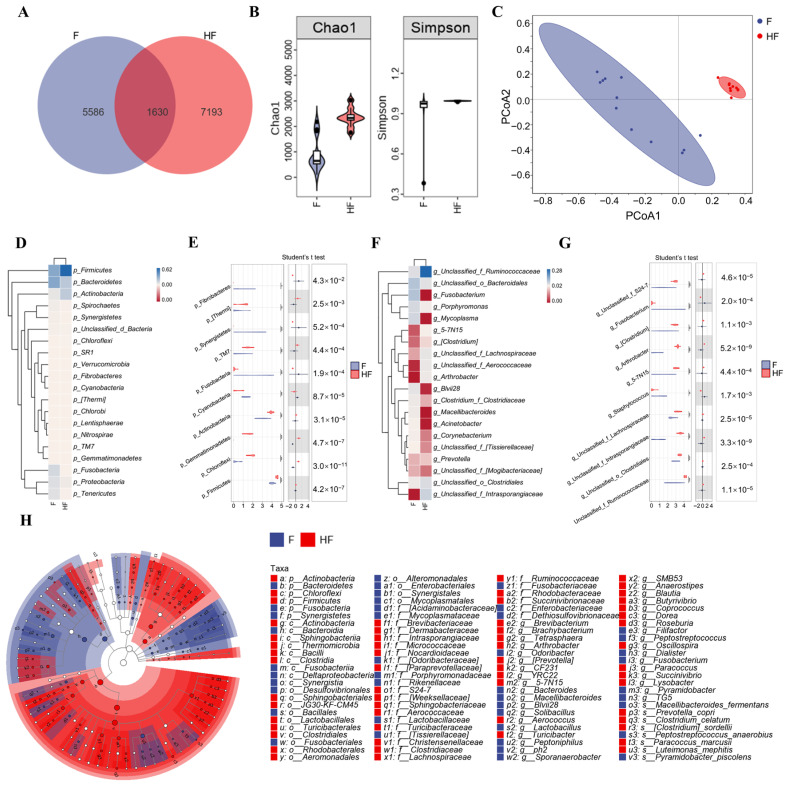
Hoof microbiota in F and HF dairy cows. (**A**) ASV counts. (**B**) Alpha diversity (Chao1 and Simpson). (**C**) PCoA based on Bray–Curtis distances. (**D**,**E**) Phylum-level composition and differential abundance. (**F**,**G**) Genus-level composition and differential abundance. (**H**) LEfSe analysis. (F = foot rot hoof samples; HF = healthy hoof samples; * *p* < 0.05, ** *p* < 0.01, and *** *p* < 0.001).

The microbial composition of hoof swabs from foot rot cows and healthy controls was analyzed using 16S rRNA gene sequencing. A total of 14,409 amplicon sequence variants (ASVs) were identified, including 8823 in the healthy group and 7216 in the foot rot group (Figure 2A). Alpha diversity indices indicated significantly lower richness in foot rot (Chao1, *p* = 3.7 × 10^−5^; Simpson, *p* = 0.0034; Figure 2B). PCoA1 and PCoA2 accounted for 30.30% and 10.6% of the total variance, respectively (PERMANOVA, R^2^ = 0.26, *p* < 0.001; Figure 2C).

At the phylum level, *Firmicutes*, *Bacteroidetes*, *Actinobacteria*, *Fusobacteria*, and *Proteobacteria* were the most abundant. Significant differences were observed in several phyla, including *Fibrobacteres*, *Thermi*, *Synergistetes*, *TM7*, *Fusobacteria*, *Cyanobacteria*, *Actinobacteria*, *Gemmatimonadetes*, *Firmicutes*, and *Chloroflexi* (Figure 2D,E). At the genus level, unclassified *Ruminococcaceae*, unclassified *Bacteroidales*, *Fusobacterium*, *Porphyromonas*, and unclassified *Clostridiales* exhibited relatively high abundances in the hoof microbiota. Unclassified *Ruminococcaceae*, unclassified *Clostridiales*, unclassified *Intrasporangiaceae*, unclassified *Lachnospiraceae*, *Staphylococcus*, *5-7N15*, *Arthrobacter*, *Clostridium*, *Fusobacterium*, and unclassified *S24-7* showed significant differences between the two groups (Figure 2F,G).

LEfSe analysis further identified *Bacteroidetes*, *Fusobacteria*, and *Synergistetes* as enriched phyla in the foot rot group, while *Firmicutes*, *Actinobacteria*, and *Chloroflexi* were dominant in healthy samples. Genera such as *Fusobacterium*, *Blvii2*, and *Macellibacteroides* were identified as potential biomarkers in the diseased group, whereas *Arthrobacter* was enriched in healthy hooves (Figure 2H). Foot rot significantly altered the hoof microbiota of dairy cows, leading to reduced diversity and distinct community composition compared with healthy animals.

### 3.3. Alterations in Milk Microbiota of Dairy Cows with Foot Rot

**Figure 3:**
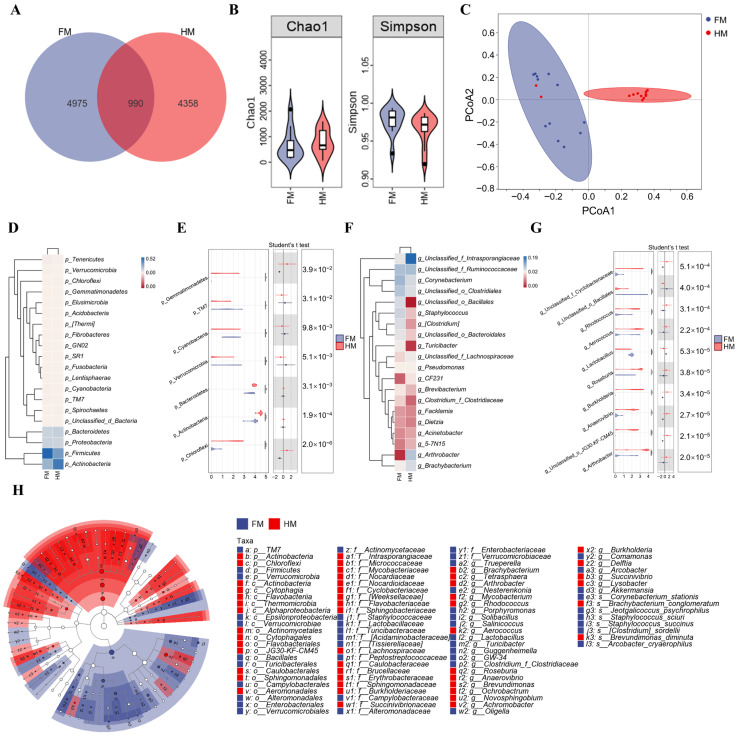
Milk microbiota in FM and HM dairy cows. (**A**) ASV counts. (**B**) Alpha diversity (Chao1 and Simpson). (**C**) PCoA based on Bray–Curtis distances. (**D**,**E**) Phylum-level composition and differential abundance. (**F**,**G**) Genus-level composition and differential abundance. (**H**) LEfSe analysis. (FM = foot rot milk samples; HM = healthy milk samples; * *p* < 0.05, ** *p* < 0.01, and *** *p* < 0.001).

A total of 10,323 ASVs were detected in milk samples, with 5965 from foot rot cows (FM) and 5348 from healthy cows (HM) (Figure 3A). Alpha diversity did not differ between FM and HM (Chao1, *p* = 0.48; Simpson, *p* = 0.13; Figure 3B). PCoA1 and PCoA2 explained 22.3% and 9.7% of the total variance respectively (PERMANOVA, R^2^ = 0.16, *p* < 0.001; Figure 3C).

*Firmicutes*, *Actinobacteria*, *Bacteroidetes*, *Proteobacteria*, and *Tenericutes* were dominant phyla. Differential abundance analysis indicated significant differences in *Gemmatimonadetes*, *TM7*, *Cyanobacteria*, *Verrucomicrobia*, *Bacteroidetes*, *Actinobacteria*, and *Chloroflexi* (Figure 3D,E). Additionally, unclassified *Intrasporangiaceae*, unclassified *Ruminococcaceae*, *Corynebacterium*, unclassified *Clostridiales*, and *Arthrobacter* exhibited relatively high abundance in the milk microbiota. At the genus level, *Arthrobacter*, *unclassified JG30-KF-CM45*, *Anaerovibrio*, *Burkholderia*, *Roseburia*, *Lactobacillus*, *Aerococcus*, *Rhodococcus*, unclassified *Bacillales*, and unclassified *Cyclobacteriaceae* showed significant differences between the two groups (Figure 3F,G).

LEfSe analysis revealed *Firmicutes*, *Verrucomicrobia*, and *TM7* as enriched phyla in FM samples. At the genus level, *Porphyromonas*, *Clostridium* and *Turicibacter* were identified as characteristic taxa in FM. In contrast, *Actinobacteria* and *Chloroflexi* were dominant in HM samples, with *Brevundimonas diminuta* and *Brachybacterium conglomeratum* identified as characteristic taxa (Figure 3H).

### 3.4. Alterations in Vaginal Microbiota of Dairy Cows with Foot Rot

**Figure 4:**
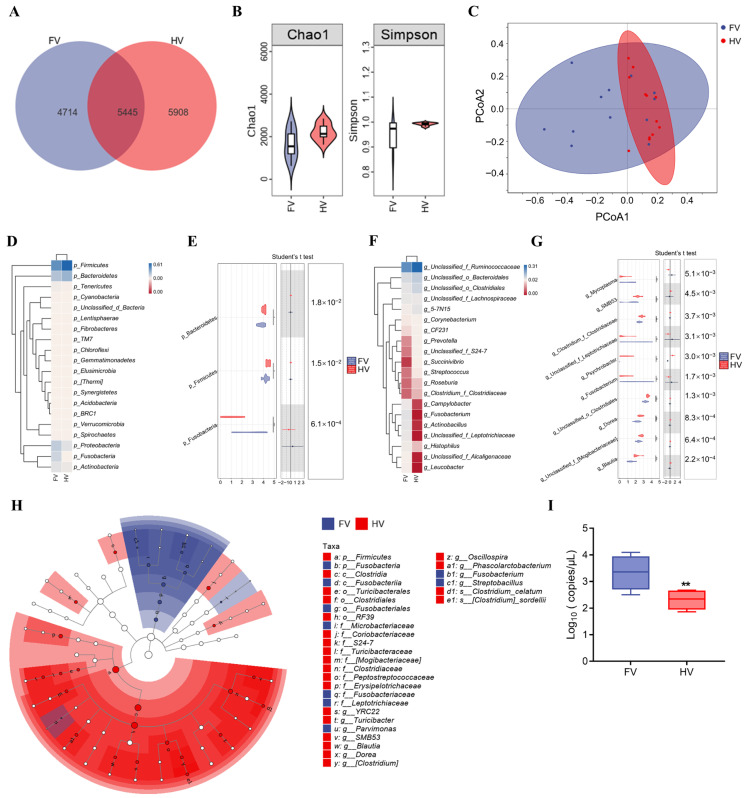
Vaginal microbiota in FV and HV dairy cows. (**A**) ASV counts. (**B**) Alpha diversity (Chao1 and Simpson). (**C**) PCoA based on Bray–Curtis distances. (**D**,**E**) Phylum-level composition and differential abundance. (**F**,**G**) Genus-level composition and differential abundance. (**H**) LEfSe analysis. (**I**) Absolute qPCR of vaginal *F. necrophorum* in FV and HV groups. (FV = foot rot vaginal samples; HV = healthy vaginal samples; * *p* < 0.05, ** *p* < 0.01, and *** *p* < 0.001).

We identified 10,622 amplicon sequence variants (ASVs), including 4714 in foot rot samples and 5908 in healthy controls (Figure 4A). Alpha diversity analysis showed that microbial richness and diversity were reduced in the FV group, with lower Chao1 (*p* = 0.068) and Simpson (*p* = 0.013) indices (Figure 4B). PCoA revealed a distinct community structure between groups. The contribution rate of PCoA1 was 15.9%, and the contribution rate of PCoA2 was 9.9% (PERMANOVA, R^2^ = 0.072, *p* < 0.001; Figure 4C).

*Firmicutes*, *Bacteroidetes*, *Proteobacteria*, *Fusobacteria*, and *Actinobacteria* were the most abundant phyla. Significant differences were observed in *Firmicutes*, *Bacteroidetes*, and *Fusobacteria* (Figure 4D,E). The main dominant genera were unclassified *Ruminococcaceae*, unclassified *Bacteroidales*, unclassified *Clostridiales*, unclassified *Lachnospiraceae*, and *5-7N15*. At the genus level, notable differences were observed in the abundance of *Blautia*, unclassified *Mogibacteriaceae*, *Dorea*, unclassified *Clostridiales*, *Fusobacterium*, unclassified *Leptotrichiaceae*, *Psychrobacter*, *Clostridium Clostridiaceae*, *Mycoplasma* and *SMB53* (Figure 4F,G).

LEfSe analysis identified *Firmicutes* as a biomarker phylum in HV samples, whereas *Fusobacteria* was enriched in FV samples. Genera such as *Fusobacterium*, *Parvimonas*, and *Streptobacillus* were characteristic of the FV group (Figure 4H). Based on the 16S rRNA gene sequencing data, *Fusobacteriaceae* was detected in the vaginal microbiota of both healthy cows and cows with foot rot, with an apparent difference in relative abundance between the two groups. Absolute qPCR analysis further confirmed significantly higher abundance of *F. necrophorum* in the FV group compared to HV (Figure 4I).

### 3.5. Foot Rot Reduces Microbial Network Connectivity and Reshapes Keystone Taxa Across Hoof, Vaginal, and Milk Microbiota

**Figure 5:**
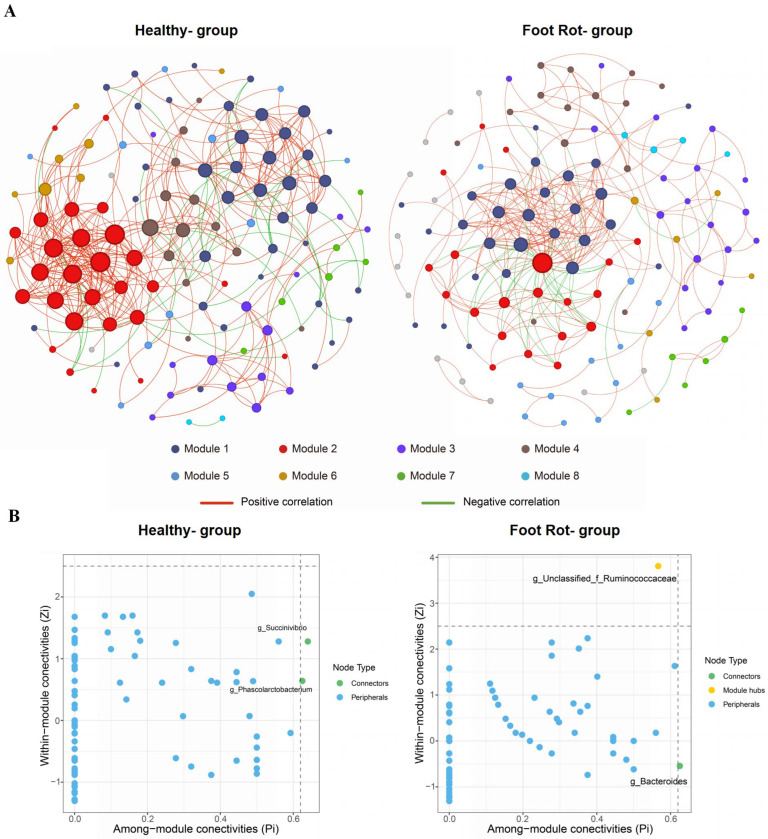
Cross-compartment co-occurrence networks. (**A**) Genus-level SparCC networks between healthy and foot rot dairy cows (|r| > 0.6, FDR < 0.05). (**B**) Zi–Pi plot showing node roles. (Only the top eight modules are highlighted in different colors, whereas the remaining modules are shown in gray).

To assess how foot rot alters the interrelationships among the hoof, vaginal, and milk microbiota in dairy cows, we constructed and compared co-occurrence networks for healthy and affected animals. In healthy cows, the network comprised 120 nodes and 402 edges, partitioned into 9 modules with a modularity value of 0.59. In cows with foot rot, the network likewise contained 120 nodes but only 299 edges, forming 14 modules with a modularity value of 0.56. In the foot rot group, the network had an average degree of 4.98, an average weighted degree of 3.31, a diameter of 16, a density of 0.04, an average clustering coefficient of 0.49, and an average path length of 5.02. In the healthy group, the corresponding values were 6.70, 4.62, 8, 0.06, 0.51, and 3.51, respectively. Relative to the healthy group, the foot rot network showed lower average degree, average weighted degree, and density; higher diameter and average path length; and a slightly lower average clustering coefficient (Figure 5A).

To pinpoint the keystone species within the co-occurrence pattern, we evaluated the topological significance of each node by considering Zi and Pi. In the healthy group, *Succinivibrio* (Pi = 0.640) and *Phascolarctobacterium* (Pi = 0.625) were identified as connectors within the co-occurrence network. In contrast, in the foot rot group, *Bacteroides* (Pi = 0.625) served as the connector, while unclassified *Ruminococcaceae* (Zi = 3.806) was identified as the module hub (Figure 5B).

## 4. Discussion

Our study revealed that dairy cows with foot rot exhibited decreased microbial diversity and altered community structure in the hoof, accompanied by significant compositional changes in the vaginal and milk microbiota. Notably, the abundance of *F. necrophorum* was significantly elevated in the vagina of affected cows. Furthermore, co-occurrence network analysis demonstrated that foot rot disrupted the ecological stability of microbial communities, resulting in reduced network complexity and connectivity. Collectively, these findings provide novel insights into the pathophysiology of foot rot, indicating that foot rot is associated with microbial alterations not only at the hoof lesion site but also in the reproductive tract and milk compartment.

Foot rot is an infectious disease that severely compromises claw health in dairy cows. It not only causes intense pain and impaired locomotion but is also accompanied by pronounced alterations in the microbial community at the lesion site. Previous studies have reported that the onset of foot rot typically reflects a shift in the hoof micro-ecosystem from a healthy, symbiotic state toward a dysbiotic, anaerobe-dominated state. This transition is characterized by a marked increase in opportunistic pathogens, particularly *Fusobacterium* and *Bacteroides*, alongside a substantial decrease in beneficial taxa associated with maintaining skin barrier function, such as members of the phylum *Actinobacteria* [21,22,23,24]. Consistent with these reports, we similarly observed a significant reduction in microbial diversity in the hooves of cows with foot rot. In addition to confirming an increased abundance of the key pathogen *Fusobacterium*, we found that *Porphyromonas* was markedly enriched in the diseased group, whereas potentially beneficial taxa such as *Arthrobacter* were significantly depleted. These shifts may have important pathological implications. Previous studies suggest that *Fusobacterium* can directly damage tissues by secreting leukotoxin and necrotizing factors, while *Porphyromonas* often acts as a synergistic pathogen by producing proteases that degrade host tissues and by consuming oxygen, thereby creating an anaerobic niche that favors *Fusobacterium* colonization [25,26]. In contrast, *Arthrobacter* is a common skin-associated commensal and may contribute to the degradation of complex organic compounds and the production of antimicrobial substances [27,28]. Its decreased abundance may therefore indicate reduced colonization resistance within the local niche. Therefore, we hypothesize that the synergistic expansion of pathogenic taxa, coupled with the depletion of protective commensals, may be associated with impaired local microbial homeostasis and potentially reduced colonization resistance. This microbial shift likely contributes to tissue necrosis and perpetuates the inflammatory response, though whether it is a cause or consequence of disease initiation remains to be determined.

Beyond the localized hoof infection, this study revealed marked dysbiosis in both the vaginal and milk microbiota of cows with foot rot. Previous studies have shown that lameness is often associated with reduced milk yield and elevated somatic cell count (SCC). This association may be partly explained by pain-related stress and behavioral changes, such as altered lying patterns, which can increase teat-end exposure to environmental bacteria. Consistent with these clinical features, we observed lower milk yield in cows with foot rot. Although the alpha diversity of the milk microbiota remained unchanged, the community structure shifted. Milk samples from cows with foot rot showed an increased relative abundance of taxa previously reported in association with mastitis, along with changes in several commensal and environmental taxa. These patterns may indicate a less resilient mammary microbial ecosystem, while functional outcomes such as epithelial barrier integrity were not directly evaluated in this study. Members of the genus *Clostridium* have been reported in association with clinical and subclinical mastitis [29,30]. Although *Porphyromonas* is not typically considered a primary mastitis pathogen, its detection in milk samples is also noteworthy [31]. Conversely, healthy cows (HM group) exhibited a higher abundance of commensal and environmental taxa, such as *Brevundimonas diminuta* and *Brachybacterium conglomeratum*, which are linked to microbial equilibrium and udder health [32,33]. Taken together, these results suggest that cows with foot rot tend to have disturbed milk microbiota, and they raise the possibility that hoof disease and mammary microbial communities may be linked.

The vaginal microbiota of cows affected by foot rot exhibited significant alterations, characterized by a notable reduction in alpha diversity and an increased presence of *Fusobacterium*, *Parvimonas*, and *Streptobacillus*. These taxa are frequently associated with anaerobic infections and reproductive tract pathologies. Notably, *Fusobacterium* has been widely recognized as a key pathogen in metritis and uterine infections [34], while *Parvimonas* have also been implicated as opportunistic pathogens in endometritis [35]. The detection of *Mycoplasma*, a bacterium linked to postpartum cytological endometritis and dystocia [36,37], further supports the association between foot lesions and reproductive tract dysbiosis. Because 16S rRNA gene sequencing showed that *Fusobacterium* was present in the vagina of both healthy and foot rot cows but at different relative abundances, we performed absolute qPCR to quantify this difference and further substantiate the increased abundance of *F. necrophorum* in the vagina of cows with foot rot. Quantitative PCR showed a higher abundance of *F. necrophorum* in the vaginal samples from cows with foot rot, which is consistent with an association between foot rot status and alterations in the vaginal microbiota. Collectively, these alterations highlight a breakdown in vaginal microbial homeostasis associated with hoof disease.

The co-occurrence network analysis indicated that foot rot was associated with reorganized microbial networks across the hoof, vaginal, and milk compartments. Although the number of nodes was comparable between healthy and affected cows, the foot rot network showed fewer edges, lower average degree and density, and greater modular partitioning, suggesting reduced cohesion of the microbial ecosystem [38]. These patterns are consistent with prior reports that disease or environmental stress can simplify and destabilize microbiome networks by lowering connectivity and cohesion and by altering mesoscale organization [39]. The increased number of modules and longer average path length further imply a loss of network integration, possibly reflecting weakened inter-compartment microbial communication or niche differentiation under disease stress. The concept of identifying keystone species via topological metrics such as Zi and Pi has been validated in environmental and livestock microbial network research [40]. The shift in keystone taxa underscores a profound restructuring of microbial roles. In healthy cows, *Succinivibrio* and *Phascolarctobacterium* acted as connectors, consistent with their roles in carbohydrate fermentation and propionate production. *Succinivibrio* correlates with ruminal propionate under starch-rich conditions, while *Phascolarctobacterium* consumes succinate to produce propionate. The epithelial benefits of short-chain fatty acids further support the protective relevance of such taxa [41,42,43]. In the foot rot network, *Bacteroides* and an unclassified *Ruminococcaceae* genus emerged as central nodes. *Bacteroides* is often linked to inflammatory contexts in dysbiosis, yet its functions are context-dependent, indicating disease-associated reorganization wherein potentially pro-inflammatory or opportunistic taxa gain structural importance [44]. It should be noted that correlation-based co-occurrence networks capture statistical association patterns and should be interpreted as hypothesis-generating rather than evidence of direct ecological interactions or causality.

Several mechanisms may contribute to the observed cross-compartmental dysbiosis. While our study did not directly trace bacterial translocation, one potential explanation that has been proposed in the literature is the hematogenous or lymphatic spread of anaerobic bacteria or their metabolic byproducts from hoof lesions to distant tissues [17]. Alternatively, foot rot might induce systemic inflammatory responses that alter mucosal immunity, thereby facilitating the colonization of opportunistic pathogens in the mammary gland and reproductive tract [45]. The rumen is the primary microbial reservoir and metabolic hub in ruminants, and it may indirectly influence extra-ruminal microbial ecology through microbial metabolites and host immunometabolic regulation. For example, rumen microbial features have been reported to be associated with mastitis in dairy cows [46], and core taxa shared between ruminal and vaginal microbiota have been identified in heifers, suggesting linked microbial signatures [47]. Therefore, the vaginal and milk microbiota differences observed in cows with foot rot in the present study may reflect shared upstream drivers associated with rumen microbial changes and their systemic regulatory effects. Future studies incorporating rumen sampling are warranted to more comprehensively evaluate the contribution of the rumen to cross-compartment microbial patterns. Additionally, behavioral or environmental factors, such as increased lying times, reduced grooming, or compromised hygiene due to lameness, may also play a role in microbial shifts. Lastly, stress-induced hormonal imbalances could disrupt immune function and microbiota homeostasis across multiple tissues. Our cross-sectional microbiome data cannot directly test these mechanisms, but they provide a basis for generating hypotheses that should be addressed in future mechanistic studies.

Our cross-compartment analysis indicates that foot rot coincides with coordinated dysbiosis in the hoof, vagina, and milk, characterized by an increase in potentially pathogenic bacteria and a loss of microbial network cohesion. Although the cross-sectional design precludes causal inference, the consistent enrichment of *F. necrophorum* in hoof lesions and its higher vaginal load in foot rot cows suggest a potential hoof–reproductive tract association that warrants further investigation. Therefore, any statements regarding systemic dissemination or downstream reproductive or mammary consequences are speculative and require longitudinal and mechanistic validation. This study also has several limitations. It was primarily designed to screen for potential associations between foot rot and alterations in the vaginal and milk microbiota, and the sample size was therefore limited rather than statistically powered to dissect detailed mechanisms. In addition, we did not measure inflammatory markers, blood microbiota, or other host physiological indices, so the pathways underlying these associations remain to be elucidated. Future studies with larger cohorts and longitudinal designs, integrating metagenomics, host immune and inflammatory markers, and contamination-controlled sampling, will be required to validate these findings and clarify the mechanisms linking hoof disease to changes in the vaginal and milk microbiota.

## 5. Conclusions

This study evaluated cross-compartment differences in the microbiota of hoof, vaginal, and milk samples from dairy cows with foot rot. The foot rot group exhibited distinct community structure across all three compartments, and the absolute abundance of vaginal *Fusobacterium necrophorum* was increased (validated by qPCR). These findings support a statistical association between foot rot and multi-compartment shifts in microbiota composition and provide a foundation for further research. Future longitudinal studies with functional validation are warranted to clarify temporal dynamics, biological significance, and potential cross-compartment links.

## Figures and Tables

**Table 1:** Baseline production and clinical characteristics of dairy cows with foot rot and healthy controls.

Parameter	Foot Rot (*n* = 12)	Healthy (*n* = 12)	*p*-Value
Parity	2.4 ± 0.5	2.6 ± 0.5	0.157
Milk yield (kg/day)	27.9 ± 1.5	33.0 ± 1.4	5.36 × 10^−6^
Days in milk (days)	133.7 ± 34.4	133.3 ± 33.7	0.774
Somatic cell count (×10^3^ cells/mL)	190.8 ± 26.4	161.4 ± 32.2	0.080
Body condition score	2.8 ± 0.7	3 ± 0.7	0.317
Rectal temperature (°C)	38.9 ± 0.4	38.6 ± 0.2	0.041

Data are presented as mean ± SD. *p* values were calculated using paired *t*-test. A *p* value < 0.05 was considered statistically significant.

## Data Availability

The raw 16S rRNA gene sequencing data have been deposited in the Genome Sequence Archive (GSA) at the National Genomics Data Center (NGDC), China National Center for Bioinformation/Beijing Institute of Genomics, Chinese Academy of Sciences, under accession CRA063243 (BioProject: PRJCA057974). The data are publicly accessible at https://ngdc.cncb.ac.cn/bioproject/browse/PRJCA057974 (accessed on 9 March 2026).
